# Blood-brain barrier impairment in MPS III patients

**DOI:** 10.1186/1471-2377-13-174

**Published:** 2013-11-13

**Authors:** Svitlana Garbuzova-Davis, Santhia Mirtyl, Sebastian A Sallot, Diana G Hernandez-Ontiveros, Edward Haller, Paul R Sanberg

**Affiliations:** 1 Center of Excellence for Aging & Brain Repair, University of South Florida, Morsani College of Medicine, 12901 Bruce B. Downs Blvd, Tampa, FL 33612, USA; 2 Department of Neurosurgery and Brain Repair, University of South Florida, Morsani College of Medicine, 12901 Bruce B. Downs Blvd, Tampa, FL 33612, USA; 3 Department of Molecular Pharmacology and Physiology, University of South Florida, Morsani College of Medicine, Tampa, FL 33612, USA; 4 Department of Pathology and Cell Biology, University of South Florida, Morsani College of Medicine, Tampa, FL 33612, USA; 5 Department of Psychiatry, University of South Florida, Morsani College of Medicine, Tampa, FL 33612, USA; 6 Department of Integrative Biology, University of South Florida, Tampa, FL 33620, USA

**Keywords:** Mucopolysaccharidosis type III, Patients, Blood-brain barrier, Endothelial cells, Pericytes, Lysosomal accumulation

## Abstract

**Background:**

Mucopolysaccharidosis type III (MPS III) is an autosomal recessive disorder caused by deficiency of a specific enzyme leading to heparan sulfate (HS) accumulation within cells and to eventual progressive cerebral and systemic organ abnormalities. Different enzyme deficiencies comprise the MPS III subcategories (A, B, C, D). Since neuropathological manifestations are common to all MPS III types, determining blood-brain barrier (BBB) condition may be critical to understand potential additional disease mechanisms.

**Methods:**

We investigated BBB integrity in various brain structures of post-mortem tissues from an eleven year old Caucasian female with MPS III A and from a twenty four year old Caucasian female with MPS III D. Control tissues were obtained post-mortem from three Caucasians without neurological deficits: a twelve year old male, a twenty four year old female, and a twenty seven year old female. BBB capillary ultrastructure (electron microscopy) and capillary functional integrity (IgG leakage, tight junction proteins, and lysosomal accumulation within endothelium) were examined.

**Results:**

Compromised BBB integrity was found in both MPS III cases. Major study findings were: (1) capillary endothelial and pericyte cell damage; (2) mucopolysaccharide bodies in a majority of endothelial cells and pericytes rupturing cell membranes; (3) severe extracellular edema; (4) IgG microvascular leakage and reductions of occludin and claudin-5 with variations between MPS III types; (5) extensive lysosomal accumulation in capillary endothelium.

**Conclusions:**

These new findings of BBB structural and functional impairment, although from only two cases, MPS III A and III D, may have implications for disease pathogenesis and should be considered in treatment development for MPS III.

## Background

Mucopolysaccharidosis type III (MPS III), or Sanfilippo Syndrome, is an autosomal recessive disorder caused by the lack of specific enzymes needed for the breakdown of heparan sulfate (HS). Different enzyme deficiencies in the HS degradation pathway comprise the four MPS III subcategories: A – heparan-*N*-sulfatase, B – α-*N*-acetylglucosaminidase, C – acetyl-CoA-*N*-acetyltransferase, and D – *N*-acetylglucosamine-6-sulfatase. The lack of any of these enzymes leads to accumulation of undegraded HS in lysosomes and eventual cell death. Although the four subcategories of MPS III are biochemically diverse, clinical manifestations are mostly indistinguishable [1-3]. However, the clinical course in MPS III A is more severe, characterized by earlier onset, more rapid symptom progression, and shorter survival [4-7] than other MPS III subtypes. Also, MPS III A and III B are the most common subtypes of MPS III; MPS III C and III D are much rarer. Patients with MPS III suffer severe cerebral and mild systemic organ abnormalities. Sanfilippo syndrome mainly affects neurons in the central nervous system (CNS), leading to delayed development, progressive mental retardation, neurological dysfunction, and severe dementia [8-10]. Death usually occurs in the patient’s second decade or by the beginning of the third decade.

Since neuropathological manifestations are common to all MPS III types, determining the blood-brain barrier (BBB) competence may be critical to identifying potential additional mechanisms of this devastating disorder. The BBB is the specialized microvasculature system within the CNS limiting entry of various substances, including pathogens, from the systemic compartment to the brain and removing toxic waste products from the brain to sustain proper neuronal function [11-15]. The primary barrier is formed by endothelial cells with tight junction protein complexes. The cerebral microvascular endothelium, in association with astrocyte end-feet, perivascular macrophages, pericytes, and basement lamina, selectively allow substances to cross into the brain. Substances with molecular weights greater than 450 Da are barred from crossing the BBB by free diffusion. Hence, structural and functional integrity of all BBB elements is essential for maintaining CNS homeostasis and any impairment of these cellular components may cause barrier breakdown.

Specific attention was made to clarify the role of the BBB in various lysosomal storage diseases, not only for the development of new therapeutic strategies for drug delivery across the barrier, but also for understanding potential BBB dysfunction’s contribution to disease neuropathology [16]. Dilatation of the perivascular space around the brain capillaries in the white matter, neuronal swelling, cortical atrophy, and ventricular enlargement have been shown in MPS III patients [10,17-20]. These findings were confirmed in a later study which also frequently demonstrated neuroimaging (CAT and MRI) anomalies such as dilated Virchow-Robin perivascular spaces, patchy changes in white matter, and ventriculomegaly in children with MPS types I, II, III, and VI [21]. In some patients with MPS I, II, or III, callosal atrophy and cerebellar changes were noted along with morphological abnormalities in Purkinje cells [22]. In the case of one MPS III A patient, cerebral atrophy, cribriform changes in the corpus callosum, basal ganglia, and white matter in addition to enlarged perivascular spaces were observed with MRI [23]. Together, these findings are suggestive as to the involvement of compromised cerebral vessels in aggravating MPS neuropathology. However, compelling evidence of BBB damage has not yet been shown in MPS III.

We recently showed BBB structural and functional impairment in a mouse model of MPS III B [24]. The MPS III B mice displayed ultrastructural abnormalities in capillary endothelia of the cortex, hippocampus, striatum, and cerebellum even at early disease stage. Vascular leakage, edematous space around microvessels, formation of large cytoplasmic vacuoles in endothelial cells and perivascular cells throughout these brain structures were noted. Accumulation of GM3 ganglioside, a secondary storage product, was determined in the brain capillary endothelium, likely leading to endothelial cell damage. These results demonstrated severe BBB breakdown which might accelerate neuronal damage. However, no data exist regarding BBB competence in humans with MPS III.

## Methods

In the present study, we examined BBB integrity in various brain structures of post-mortem tissues from two patients, one with MPS III A and one with MPS III D. We determined not only substantial structural BBB impairment in both cases, but also functional CNS barrier damage, which may have implications for disease pathogenesis. These new findings should be considered as a basis for determining the role of vascular dysfunction in MPS III and, specifically, are supportive of targeting the BBB for treatment development.

Frozen post-mortem hippocampus, cerebellum, putamen, and primary motor cortex tissues from patients with MPS III A (female, 11 years old) and MPS III D (female, 24 years old), along with age-matched control tissues without neurological pathologies were received from the National Institute of Child Health and Development (NICHD) Brain and Tissue Bank for Development Disorders at the University of Maryland, Baltimore, MD. Autolysis times for tissues from MPS III patients and controls were between 6 and 19 hours (average, 12.8 hours). Summaries of health histories for each patient and control individual were provided by the tissue bank.

Brain tissues were prepared immediately upon arrival for electron microscope, Western blot, or immunohistochemical analysis as described [25]. Briefly, for electron microscopy (EM), tissue samples were fixed in 4% paraformaldehyde (PFA) in 0.1 M phosphate buffer (PB), pH 7.2, for 16-24 hours at 4°C. Next day, tissues were cut into 1 mm slices and fixed overnight in 2.5% glutaraldehyde in 0.1 M PB (Electron Microscopy Sciences, Inc., Hatfield, PA) at 4°C. The following day, tissues were transferred to a fresh change of buffer and stored for further EM processing. For Western blot analysis, part of each brain tissue was dissected and stored at -80°C for later analysis of tight junction proteins. For immunohistochemistry, tissues were fixed in 4% PFA in 0.1 M phosphate buffered saline (PBS) for 24 hours. Brain tissues were then cryoprotected in sucrose solution (20% sucrose in 0.1 M PBS, pH 7.2) for 48 hours at 4°C. Coronal brain tissue sections were cut at 30 μm in a cryostat, maintained on slides and stored at -20°C.

BBB ultrastructural integrity was characterized in various brain regions of MPS III patients and compared to age-matched controls by electron microscopy as described previously [24,25]. Briefly, tissue samples were post-fixed in 1% osmium tetroxide (Electron Microscopy Sciences, Inc., Hatfield, PA) and dehydrated in acetone. After fixation and dehydration, brain tissues were infiltrated with LX112 epoxy resin embedding mix (Ladd Research Industries, Burlington VT) and placed in molds to polymerize. Blocks were polymerized at 70°C in an oven overnight and then trimmed and sectioned with a diamond knife on an LKB Huxley ultramicrotome. Thick sections cut at 0.35 μm were placed on glass slides and stained with 1% toluidine blue stain. Thin sections were cut at 80-90 nm, placed on copper grids, and stained with uranyl acetate and lead citrate. Tissue sections were examined and photographed with a FEI Morgagni transmission electron microscope (FEI, Inc., Hillsboro, OR) and Olympus MegaView III digital camera (ResAlta Research Technologies Corp., Golden, CO.) at 60 kV.

Western blot assay was performed as we previously described [25]. Briefly, tissue samples were homogenized in 1× cell lysis buffer (Cell Signaling Technology) with 1% protease inhibitor cocktail (Sigma-Aldrich) and centrifuged at 10,000×*g* during 60 min at 4°C. Electrophoresis gels (4-15% Mini Protean TGX, Bio-Rad) were loaded with 20 μg of total protein per sample per well, and current applied at 90 V, during 90 min. Proteins were transferred to nitrocellulose membranes (Bio-Rad), which were incubated overnight with one of the following primary antibodies: rabbit polyclonal occludin (1:2000, Abcam) or rabbit monoclonal claudin-5 (1:750, Abcam). The next day, membranes were incubated with the corresponding secondary antibody conjugated with horseradish peroxidase (HRP): goat anti-rabbit (1:5,000, Abcam) or rabbit anti-mouse (1:5,000, Abcam). After the addition of HRP substrate (Immobilion Western, Millipore) membranes were placed into a Bio-Rad CCD camera for band chemiluminescence detection. Images were analyzed through Quantity One software (Bio-Rad) for band density measures. Glyceraldehyde 3-phosphate dehydrogenase (rabbit anti-GAPDH, 1:10,000, Sigma-Aldrich) was used as a normalizing protein in all membranes. For each evaluated protein, the ratio between the band densities and GAPDH was established.

Immunohistochemical staining was performed to determine vascular integrity in the brain tissues from MPS III patients compared to controls as described [25]. For capillary leakage, tissue sections were incubated in a blocking solution (10% normal goat serum and 0.3% Triton X-100 in PBS) for 60 min at room temperature (RT). Then, goat anti-human IgG conjugated with FITC (1:400, Alpha Diagnostics) was applied on tissues for 2 hrs at RT. After washing, slides were coverslipped with Vectashield containing DAPI. Tissue examination and imaging were performed using an epifluorescence microscope (Olympus BX40). A second set of tissue sections was used for examination of lysosomal accumulation within endothelial cells of brain structures. A double staining for lysosomes and microvascular collagen IV was performed. Brain tissues were blocked in a pre-incubation solution as noted above for 60 min. Primary rabbit polyclonal anti-collagen IV antibody (1:500, Abcam) was applied to the tissue and incubated overnight at 4°C. The following day, tissues were incubated with goat anti-rabbit secondary antibody (1:800, Vector Labs) for 2 hrs at RT. After several washes, primary mouse monoclonal LAMP-1 antibody (1:150, Abcam) was applied to the tissues overnight at 4°C. The next day, secondary goat anti-mouse antibody (1:500, Vector Labs) was applied to tissues for 2 hours at RT. Slides were coverslipped with DAPI (Vector Labs) and tissues were examined using an epifluorescence microscope (Olympus BX40). Semi-quantitative analysis of LAMP-1 immunoexpression within capillary endothelium in all analyzed brain structures was performed. Tissue images (n = 3-5/brain structure) were assessed according to a three-point scale: 1 point was assigned to baseline control level; 2 points for noticeable immunoreactivity increase; 3 points for intensive immunoreactivity increase.

## Results

A brief summary of the health history for each patient was provided by NICHD Brain and Tissue Bank. *MPS III A patient:* female, Caucasian, 11 years old. The deceased was the only one affected of four children. The disease duration was 5 years and progressed rapidly. Two years before death, the patient was attending school although she had trouble walking and eating. The eating problems worsened. At the time of death, the patient was non-ambulatory and non-verbal with deteriorating psychomotor skills and self-injurious behavior. She was taking haldol 2 mg, b.i.d., for problems with behavior, sleep and agitation. The patient also suffered from mitral valve prolapse with myxomatous changes and mild regurgitation. *MPS III D patient:* female, Caucasian, 24 years old. The patient was one of two siblings suffering from MPS III. She suffered progressive neurologic decline with loss of neurocognitive functions including reading, verbal expression, vision, and continence. She had rare seizures despite anticonvulsive treatment. The patient was wheelchair bound for the last year of life. Neuropathologic diagnosis: generalized cerebral atrophy and neuronal storage disorder, consistent with the clinical diagnosis of MPS III D. *Control subjects:* male (Caucasian, 12 years old), female (Caucasian, 27 years old), and female (Caucasian, 24 years old). Causes of death were accidental (drowning and motor vehicle accident) or respiratory failure. Neuropathologic examinations indicated no gross or microscopic findings in the brains.

Ultrastructural integrity of the vessels in various brain structures was examined in post-mortem tissues from MPS III A and III D patients and controls using electron microscopy.

### MPS III A patient

**Figure 1:**
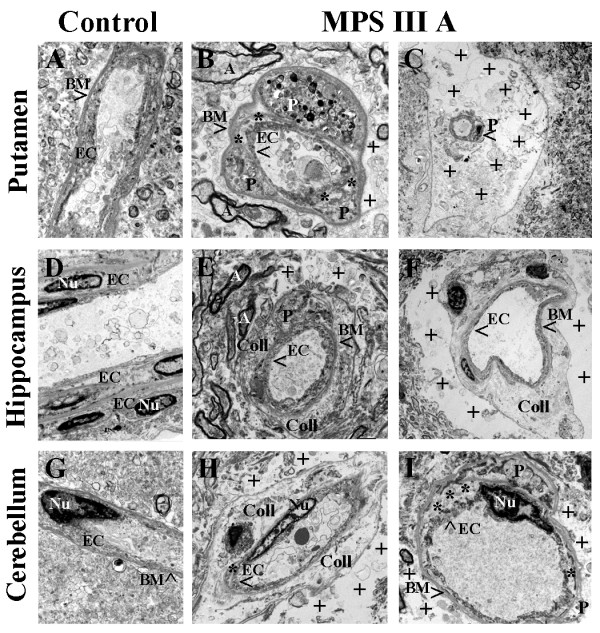
**Electron microscope examination of microvasculature in the brain from MPS III A patient.** Representative capillaries in *putamen ***(A)**, *hippocampus ***(D)**, and *cerebellum ***(G) **of the control brain were characterized by normal ultrastructural appearance. In MPS III A patient, capillaries in the *putamen ***(B, C)**, *hippocampus ***(E, F)**, and *cerebellum ***(H, I) **showed swollen and degenerated EC and pericytes containing mucopolysaccharides. Significant protein-filled perivascular spaces surrounding a capillary were observed in all analyzed brain structures. A large accumulation of disorganized collagen was observed between BM covering the ECs **(E, F, H)**. Some capillaries displayed ECs separating from the BM **(B, H, I)**. EC - endothelial cell, BM - basement membrane, **A **– axon, **P **– pericyte, **Nu **– nucleus, **Coll **– collagen accumulation, * - separation of EC from BM or P, **+ **- extracellular edema. Magnification in **(A), (E) **is 5,600; in **(B) **is 7,100; in **(C)**, **(F)**, is 1,800; in **(D), (G) **is 3,500; in **(H) **is 2,800; in **(I) **is 4,400.

Electron microscope examination of microvasculature in the control brain for MPS III A revealed normal ultrastructural appearance in *putamen* (Figure 1A), *hippocampus* (Figure 1D), and *cerebellum* (Figure 1G) capillaries consisting of endothelial cells (EC) and tight junctions, surrounded by a single layer of basement membrane (BM), astrocytes, neuropil, and myelinated nerve fibers. Organelles in all cells were well formed. In MPS III A patient, a capillary in the *putamen* showed (Figure 1B) a swollen EC, pericyte cell degeneration and accumulation of mucopolysaccharides in the space formerly occupied by a pericyte, trapped by basement membrane; edema was apparent between pericytes. A large protein-filled perivascular space surrounding a capillary, which contained free-floating swollen mitochondria, was observed (Figure 1C). Many myelinated nerves had degenerated. A capillary in the *hippocampus* demonstrated complete pericyte degeneration (Figure 1E) and pericyte fragments were noted in the adjacent extracellular space under the BM (Figure 1F). Significant edema was evident outside the capillary (Figure 1F). A large accumulation of disorganized collagen was observed between BM covering the ECs in the hippocampus (Figure 1E,F). In the *cerebellum* of MPS III A patient, collagen accumulation surrounding capillary (Figure 1H) and pericyte degeneration (Figure 1I) were also observed in addition to extracellular edema. Some capillaries displayed ECs separating from the basement membrane (Figure 1I).

### MPS III D patient

**Figure 2:**
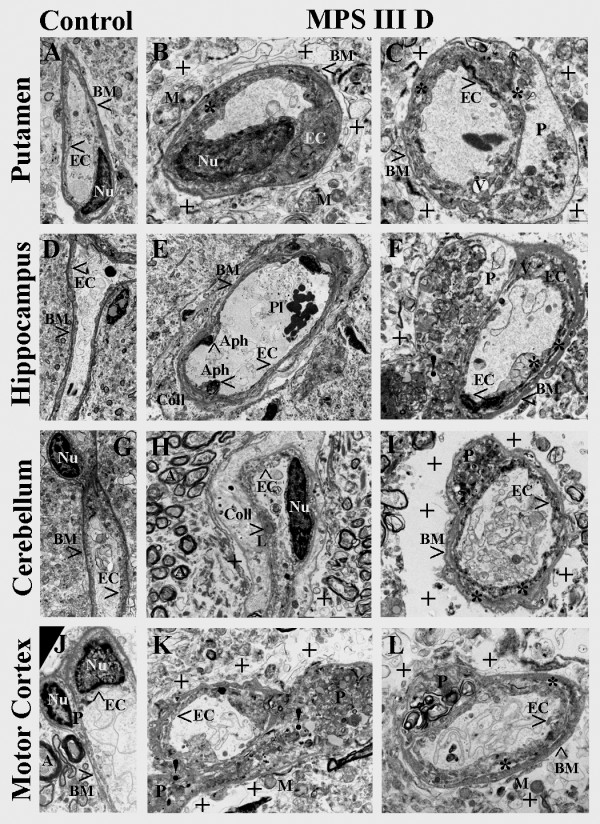
**Electron microscope examination of microvasculature in the brain from MPS III D patient.** Representative longitudinal capillaries of control *putamen ***(A)**, *hippocampus***(D)**, *cerebellum ***(G),** and *motor cortex ***(J) **were characterized by normal ultrastructural appearance indicating intact BBB. In MPS III D patient, capillaries in the *putamen ***(B, C)**, *hippocampus ***(E, F)**, *cerebellum ***(H, I), **and *motor cortex ***(K, L) **showed swollen EC narrowing the lumen or necrotic EC separating from the BM. Completely degenerated pericyte, perivascular edema, and vacuoles or autophagosomes in EC were also observed. Some capillaries displayed ruptured degenerated pericytes releasing zebra bodies into extracellular space **(F, K)**. Capillaries also showed large collagen accumulations separating the BM **(E, H) **and swollen endothelium with lipid inclusion **(H)**. Large areas of extracellular edema were observed surrounding capillaries and free floating swollen mitochondria in the space occupied by degenerated astrocyte foot processes. **EC **- endothelial cell, **BM **- basement membrane, **A** – axon, **P **– pericyte, **Nu** – nucleus, **M **– mitochondrion, **V** – vacuole, **Aph **– autophagosome, **Pl** – platelets, **Coll **– collagen accumulation, **L **– lipid inclusion, * - separation of EC from BM or P, **+ **- extracellular edema, **! **– ruptured pericyte. Magnification in **(A), (G)** is 3,500; in **(B), (C), (L) **is 8,900; in **(D) **is 2,200; in **(E) **is 1,800; in **(F), (I), (K) **is 7,100; in **(H) **is 5,600; in **(J) **is 4,400.

Capillaries in control brain tissues for MPS III D: *putamen* (Figure 2A), *hippocampus* (Figure 2D), *cerebellum* (Figure 2G), and *primary motor cortex* (Figure 2J) displayed normal ultrastructure. A single layer of endothelial cells is surrounded by a single layer of BM, forming an intact BBB. Astrocyte cell processes were adjacent to the outer surface of the capillary BM. In the *putamen* of the MPS III D patient, a capillary showed swollen EC narrowing the lumen (Figure 2B) and necrotic EC separating from the BM (Figure 2C). Completely degenerated pericyte, perivascular edema and vacuoles in EC were also observed (Figure 2C). Extracellular edema was evident (Figure 2B,C). In the *hippocampus*, the EC contained large autophagosomes (Figure 2E). Another capillary displayed a ruptured degenerated pericyte, containing mucopolysaccharides, and necrotic EC (Figure 2F). A vacuole occupied part of the cell’s cytoplasm. A capillary in *cerebellum* also showed large collagen accumulation separating the BM and swollen endothelium with lipid inclusion (Figure 2H). EC appeared to be completely separated from the BM in another capillary and degenerated pericyte, filled with mucopolysaccharide bodies, was evident (Figure 2I). Large areas of extracellular edema were observed surrounding this capillary. In the *primary motor cortex*, a ruptured pericyte releasing zebra body into extracellular space was determined (Figure 2K,L). ECs were swollen or separated from the BM. There was also evidence of extracellular edema and free floating swollen mitochondria in the space occupied by degenerated astrocyte foot processes.

In summary, capillary ultrastructural abnormalities in various brain structures were evident in post-mortem tissues from MPS III A and III D patients. Detected vascular endothelium damage including pericyte degeneration led to severe BBB impairment.

**Figure 3:**
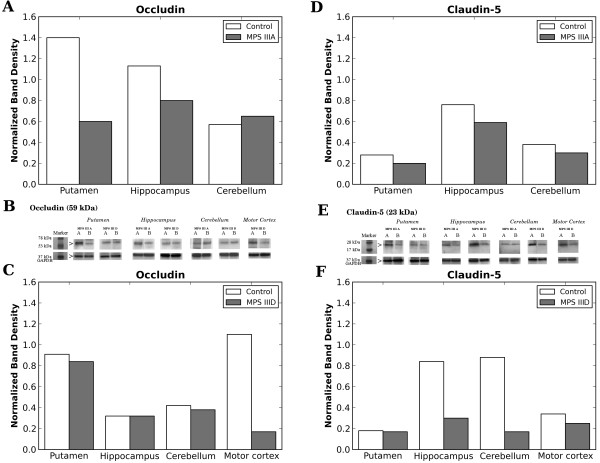
**Western blot analysis of tight junction protein expressions in various brain structures from MPS III patients. (A) **In MPS III A patient, occludin expression was decreased in both putamen and hippocampus, whereas there were no differences in cerebellum compared to control. **(D) **For claudin-5, slight decrease of protein expression was determined in all three CNS structures evaluated. In MPS III D patient, significant decrease of occludin expression was seen only in primary motor cortex **(C) **and claudin-5 – in hippocampus and cerebellum **(F)**. Bands for occludin **(B) **and claudin-5 **(E) **expressions in various brain structures of MPS III A and MPS III D patients and controls are presented. **A **columns within panels B and E are bands for control, **B **columns within panels B and E are bands for MPS III A or III D. GAPDH was used as a normalizing protein.

Tight junction (occludin and claudin-5) proteins were analyzed in various brain areas from MPS III patients and controls using Western blot. Results demonstrated downregulation of occludin expression in both putamen and hippocampus in MPS III A, whereas there were no differences in cerebellum compared to control (Figure 3A,B). For claudin-5, a slight decrease of protein expression was determined in all three CNS structures evaluated (Figure 3D,E). In brain tissues from the MPS III D patient, a major decrease of occludin expression was seen only in primary motor cortex (Figure 3C,B) and claudin-5 – in hippocampus and cerebellum (Figure 3F,E).

Differences in reductions of tight junction protein expressions in brain structures were determined between MPS III A and III D. Occludin expression was mainly reduced in putamen and hippocampus of MPS III A patient and in primary motor cortex of MPS III D patient. Although lessened expression of claudin-5 was established in all analyzed brain structures in MPS III A patient, a more pronounced decline in expression of this protein was determined in hippocampus and cerebellum from MPS III D patient.

**Figure 4:**
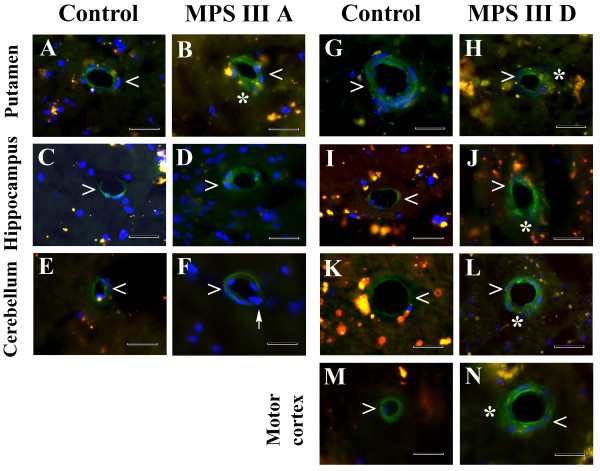
**Detection of endogenous IgG immunofluorescence in various brain structures from MPS III patients.** In control tissues for MPS III A **(A, C, E) **and MPS III D **(G, I, K, M)**, IgG was limited to within the capillary lumen. **(B) **In MPS III A tissues, IgG was detected on the abluminal side of capillaries in the putamen (green, asterisk). No vascular leakage for IgG was identified in hippocampus **(D) **or cerebellum **(F) **of this patient. Possible extravasation of leukocytes through vascular wall as indicated by DAPI nuclear staining was seen in cerebellum (**F**, arrow). In contrast, numerous capillaries in all analyzed brain structures of MPS III D patient **(H, J, L, N) **appeared blurry; IgG was dispersed into CNS parenchyma (green, asterisks) indicating cerebral microvascular leakage. Arrowhead – capillary, asterisk – vascular leakage. Scale bar in **A-N **is 25 μm.

Immunohistochemical analysis for microvascular integrity in various brain structures in MPS III patients was performed by IgG staining. In control tissues for MPS III A (Figure 4A,C,E) or MPS III D (Figure 4G,I,K,M), IgG was clearly detected within the capillary lumen. In MPS III A tissues, IgG was detected on the abluminal side of capillaries in the putamen (Figure 4B). Although no vascular leakage for IgG was identified in hippocampus or cerebellum of this patient, possible extravasation of leukocytes through vascular wall as indicated by DAPI nuclear staining was seen in cerebellum (Figure 4F). In contrast, numerous capillaries in all analyzed brain structures of MPS III D patient appeared blurry; IgG was dispersed into CNS parenchyma (Figure 4H,J,L,N) indicating pervasive cerebral microvascular leakage.

**Figure 5:**
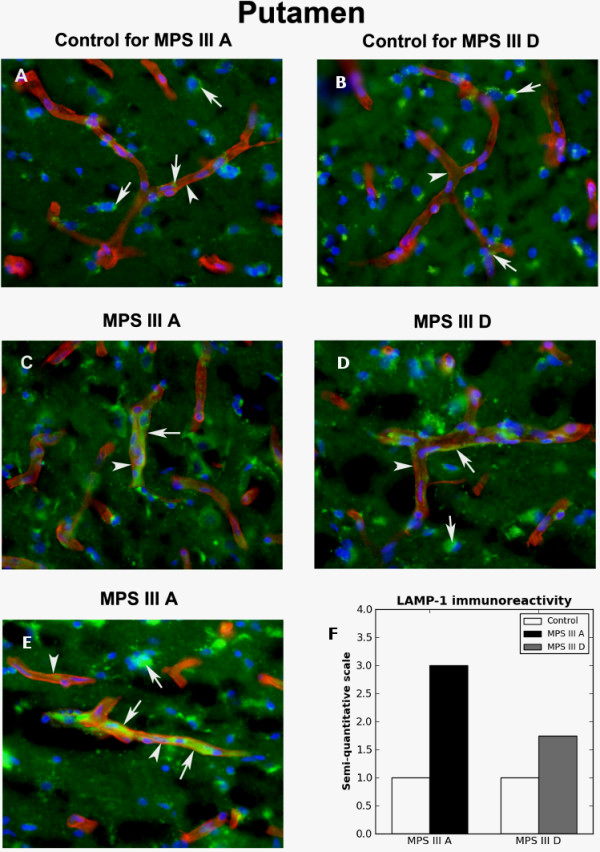
**Immunohistochemical staining for lysosomes within capillary endothelium in putamen of MPS III patients. **In putamen capillaries of control tissues **(A, B)**, evident by expression of the basement membrane collagen IV (arrowhead, red), only minor lysosomal immunoexpression of LAMP-1 (arrows, green) was seen in vessel endothelium. **(C, E) **High density of LAMP-1 immunoreactivity (arrows, green) was found within MPS III A capillaries (arrowheads, red), along with swelling of endothelial cells. **(D) **Elevated, but less severe than in MPS III A, LAMP-1 immunoexpression in capillary endothelium was detected in MPS III D putamen (arrow, green). **(D, E) **Also, increased LAMP-1 immunoreactivity was identified in neural cells from both MPS III cases. Arrows and arrowheads indicate LAMP-1 (green) and collagen IV (red) immunoexpressions, respectively. Magnification in **A-E **is 40×. **(F) **Semi-quantitative analysis of LAMP-1 immunoreactivity showed higher lysosomal marker expression within capillary endothelium in MPS III A putamen vs. III D.

Typical diminutive lysosomal immunoexpression of LAMP-1 was seen within vessel endothelium and neural cells in putamen of control tissues (Figure 5A,B). Expression of basement membrane collagen IV was clear along the margin of the vascular wall. However, high lysosome immunoreactivity was determined in MPS III A putamen capillaries along with swelling of ECs (Figure 5C,E,F). Lower, but still elevated, reactivity of lysosomes in capillary endothelium was detected in MPS III D putamen (Figure 5D,F). Also, increased LAMP-1 immunoreactivity was identified in neural cells from both MPS III cases.

**Figure 6:**
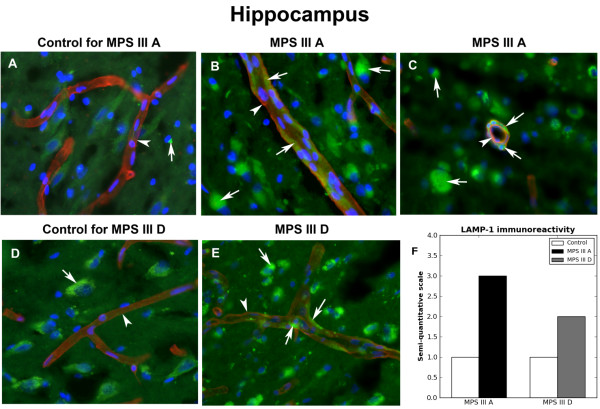
**Immunohistochemical staining for lysosomes within capillary endothelium in hippocampus of MPS III patients. **Similar to immunoexpression of lysosomes in control putamen capillaries, almost no capillary LAMP-1 expression (green) was distinguished in control hippocampus (**A, D**, arrows). Normal appearance of morphologically determined neural cells with nuclei centered within cell bodies and some lysosomal expression was demonstrated. In MPS III A **(B, C) **and III D **(E)**, intensive lysosomal LAMP-1 expression was found in numerous capillaries in hippocampus with visible swelling of ECs. **(B, C, E) **Severe LAMP-1 immunoreactivity (green, arrows) in numerous neural cells with translocation of nuclei was observed. Arrows and arrowheads indicate LAMP-1 (green) and collagen IV (red) immunoexpressions, respectively. Magnification in **A-E** is 40x. **(F) **Semi-quantitative analysis of LAMP-1 immunoreactivity showed high lysosomal marker expressions within capillary endothelium in MPS III A and III D hippocampus vs. controls.

Similar to immunoexpression of lysosomes in control putamen capillaries, almost no capillary LAMP-1 expression was distinguished in control hippocampus (Figure 6A,D). Normal appearance of morphologically determined neural cells with nuclei centered within cell bodies and some lysosomal expression was demonstrated. In MPS III A (Figure 6B,C,F) and III D (Figure 6E,F), intensive lysosomal expression was found in numerous capillaries in hippocampus with visible swelling of ECs. Severe LAMP-1 immunoreactivity in numbers of neural cells with translocation of nuclei was observed.

**Figure 7:**
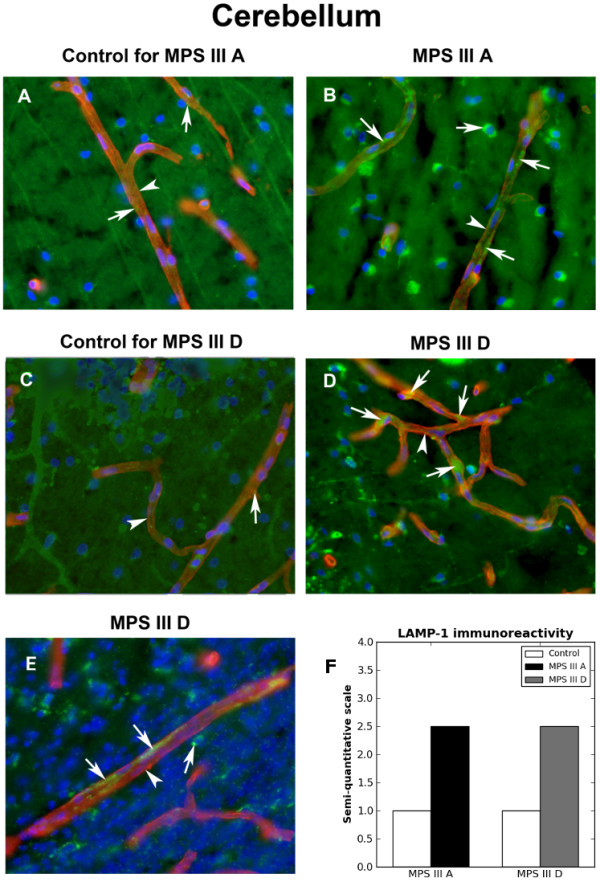
**Immunohistochemical staining for lysosomes within capillary endothelium in cerebellum of MPS III patients. (A, C) **Cerebellum control tissues contained capillaries with little lysosomal immunoexpression (arrows, green). A morphologically normal Purkinje cell with its dendrites was seen **(C)**. Increasing LAMP-1 immunoreactivity within capillary endothelium was determined in cerebellum of **(B) **MPS III A (green, arrows) and **(D, E) **MPS III D (green, arrows). Arrows and arrowheads indicate LAMP-1 (green) and collagen IV (red) immunoexpressions, respectively. Magnification in **A-E **is 40x. **(F) **Semi-quantitative analysis of LAMP-1 immunoreactivity showed similar high lysosomal marker expressions within capillary endothelium in MPS III A and III D cerebellums vs. controls.

Cerebellum control tissues contained capillaries with little lysosomal immunoexpression (Figure 7A,C). A morphologically normal Purkinje cell with its dendrites was seen. Increasing LAMP-1 immunoreactivity within capillary endothelium was determined in cerebellum of MPS III A (Figure 7B,F) and III D (Figure 7D,E,F). Of note, similar high lysosomal immunoexpression was demonstrated in capillary endothelium in MPS III D primary motor cortex.

## Discussion

In the present study, we investigated BBB integrity in various brain structures (putamen, hippocampus, cerebellum, and primary motor cortex) of post-mortem tissues from patients with MPS III A and III D. Major findings of our study were: (1) endothelial and pericyte cell degeneration; (2) endothelial and pericyte cells with numerous cytoplasmic vacuoles; (3) mucopolysaccharide bodies in a majority of endothelial cells and pericytes rupturing cell membranes; (4) severe intra- and extracellular edema; (5) significant accumulation of basement membrane collagen IV; (6) IgG microvascular leakage; (7) reductions of occludin and claudin-5 with variations between MPS III types; (8) extensive lysosomal accumulation in capillary endothelium. These new findings of BBB structural and functional impairment in various brain structures, although from only two reported cases, MPS III A and MPS III D, may have implications for disease pathogenesis and should be considered in treatment development for MPS III.

The BBB is a highly integrated microvascular barrier regulating the CNS environment. Structural and functional BBB integrity is essential for influx of required substances by specific transport systems and efflux of cellular waste products [13,15,26]. A damaged BBB would likely lead to impaired influx and/or efflux systems and a deteriorating CNS.

In our two MPS III A (the most common Sanfilippo subtype) and MPS III D (the rarest Sanfilippo subtype) cases, similar endothelial and pericyte cell degeneration due to undegraded products leading to cell membrane rupture is one of the major findings. Degeneration and even necrotic changes of these cells due to formation of vacuoles, containing mucopolysaccharides, in different brain structure capillaries could significantly contribute to BBB impairment accelerating neuronal pathology and neurological dysfunction. This microvascular damage in both reported MPS III cases was confirmed by extensive lysosomal accumulation within the endothelium. We recently demonstrated significant accumulation of GM3 ganglioside in numerous cerebral capillaries of MPS III B mice even at early disease stage, leading us to consider that accumulated storage products are possible primary effectors damaging endothelial cells [24]. It is important to emphasize that damage to endothelial cells and pericytes by the uncatabolized substrates could be comparable with neuronal secondary metabolic perturbations induced by accumulation of HS [9,27]. Also, since normal maintenance of vessels depends on interactions between pericytes and ECs via various secreted soluble factors [28,29], damage to these cells might severely compromise BBB integrity.

Common to both MPS III cases was also significant disorganized collagen accumulation within basement membrane in numerous cerebral capillaries. Collagen, specifically collagen type IV, along with laminin, heparan sulfate proteoglycans, and entactin, is a major basement membrane component [30] not only supporting the abluminal surface of the endothelium, but also restricting passage of macromolecules and cells through the BBB [31]. Although the cause of basement membrane collagen deposition is still unknown, it is possible that matrix metalloproteinases (MMPs), expressed by endothelial cells, neurons, and glial cells to degrade components of the extracellular matrix [32-34], are defectively involved in degradation of this basement membrane component due to damaged endothelial cells and neurons in MPS III. Also, a potential imbalance between MMPs and tissue inhibitors of metalloproteinases (TIMPs) in MPS III may lead to either extracellular matrix breakdown or to excessive collagen deposition as has been shown in cerebral ischemia [35]. However, further investigations are necessary to clarify the role of MMPs/TIMPs in basement membrane collage accumulation.

Despite similar structural capillary abnormalities in two MPS III cases, functional BBB impairment differed. Differences in reductions of tight junction occludin and claudin-5 protein expressions in the brain structures were determined between MPS III A and III D. The largest reduction of occludin expression was demonstrated in putamen and hippocampus of MPS III A patient and in primary motor cortex of MPS III D patient. A slight decrease of claudin-5 was established in all analyzed MPS III A brain structures. In MPS III D patient, the largest reductions in expression of this protein were determined in hippocampus and cerebellum. Currently, no definitive explanation can be made for this discrepancy. Also, since primary motor cortex tissue was limited to only the MPS III D case, comparative analysis of tight junction proteins for this brain structure was not possible. One of our speculations is that the observed differences in downexpression of occludin and claudin-5 might reflect the relative severity of neuropathological changes in these CNS regions between MPS III A and III D. The MPS III A patient suffered from progressive deterioration of psychomotor skills and died at 11 years of age. The MPS III D patient had progressive decline of neurologic and neurocognitive function and age of death was 24. Additionally, supporting our suggestion, we observed differences in vascular leakage for IgG in brain structures of MPS III patients. Capillary leakage was determined mostly in putamen from MPS III A, whereas numerous capillaries in all analyzed brain structures of MPS III D patient appeared leaky. While the tight junctions comprise a group of proteins controlling the paracellular passage of molecules [36,37], additional investigation are needed to determine expressions of the zonula occludens and cingulin proteins, which bind to actin, a cytoskeleton protein responsible for endothelial cell structural support [37,38].

## Conclusions

In summary, our study results demonstrate BBB structural and functional impairment in various brain structures in MPS III A and MPS III D cases. The BBB impairment may have implications for disease pathogenesis and should be considered in treatment development for MPS III, particularly in regards to drug delivery across the CNS barrier. Special attention should be given to endothelial cell function in view of possible deterioration of influx/efflux transport systems in maintaining proper CNS homeostasis. Restoration of BBB integrity might benefit MPS III patients by re-establishing vascular function and neuroprotection.

## Competing interests

The authors declare that they have no competing interests.

## Authors’ contributions

SGD, was responsible for study design, data analysis and writing the manuscript. SM performed immunohistochemistry and Western blot assay. SS performed immunohistochemistry for lysosomal accumulation. DHO performed immunohistochemistry and Western blot assay and analyzed data. EH performed electron microscopy, analyzed images, and wrote image descriptions. PRS was involved in study design and discussion of results. All authors read and approved the final manuscript.

## Pre-publication history

The pre-publication history for this paper can be accessed here:

http://www.biomedcentral.com/1471-2377/13/174/prepub
